# Evaluation of the long-term effect of polyhexamethylene guanidine phosphate in a rat lung model using conventional chest computed tomography with histopathologic analysis

**DOI:** 10.1371/journal.pone.0256756

**Published:** 2021-09-07

**Authors:** Cherry Kim, Sang Hoon Jeong, Jaeyoung Kim, Ja Young Kang, Yoon Jeong Nam, Ariunaa Togloom, Jaehyung Cha, Ki Yeol Lee, Chang Hyun Lee, Eun-Kee Park, Ju-Han Lee

**Affiliations:** 1 Department of Radiology, Ansan Hospital, Korea University College of Medicine, Danwon-gu, Ansan-si, Gyeonggi, South Korea; 2 Medical Science Research Center, Ansan Hospital, Korea University College of Medicine, Danwon-gu, Ansan-si, Gyeonggi, South Korea; 3 Department of Radiology, College of Medicine, Seoul National University, Seoul National University Hospital, Seoul, South Korea; 4 Department of Medical Humanities and Social Medicine, College of Medicine, Kosin University, Busan, South Korea; 5 Department of Pathology, Ansan Hospital, Korea University College of Medicine, Danwon-gu, Ansan-si, Gyeonggi, South Korea; Medical University of Graz, AUSTRIA

## Abstract

There have been no studies on the effects of polyhexamethylene guanidine phosphate (PHMG) after a long period of exposure in the rodent model. We aimed to evaluate long-term lung damage after PHMG exposure using conventional chest computed tomography (CT) and histopathologic analysis in a rat model. A PHMG solution was intratracheally administrated to 24 male rats. At 8, 26, and 52 weeks after PHMG instillation, conventional chest CT was performed in all rats and both lungs were extracted for histopathologic evaluation. At 52 weeks after PHMG instillation, four carcinomas had developed in three of the eight rats (37.5%). Bronchiolo-alveolar hyperplasia and adenoma were found in rats at 8, 26, and 52 weeks post-instillation. The number of bronchiolo-alveolar hyperplasia significantly increased over time (P-value for trend< 0.001). The severity of lung fibrosis and fibrosis scores significantly increased over time (P-values for trend = 0.002 and 0.023, respectively). Conventional chest CT analysis showed that bronchiectasis and linear density scores suggestive of fibrosis significantly increased over time (P-value for trend < 0.001). Our study revealed that one instillation of PHMG in a rat model resulted in lung carcinomas and progressive and irreversible fibrosis one year later based on conventional chest CT and histopathologic analysis. PHMG may be a lung carcinogen in the rat model.

## Introduction

From 2006 to 2011, an epidemic of interstitial lung disease occurred in South Korea owing to inhalation exposure to toxic chemicals in humidifier disinfectants (HD) [1–3]. Since their commercial introduction, an estimated eight million people have been exposed to humidifier disinfectant-chemicals that are added to humidifier water to prevent microorganism growth [1]. The major component involved was polyhexamethylene guanidine phosphate (PHMG) [1,2], well known to induce lung fibrosis and inflammation in the rodent model [4–9]. However, previous rodent model PHMG experiments were for less than 10 weeks after PHMG exposure and the authors mainly focused on acute lung disease. There is no research on the long-term effects of PHMG after exposure for more than 10 weeks in the rodent model. Approximately 30% of Korean children were exposed to PHMG-containing humidifier disinfectants [2]. Given the age of these children, a follow-up period of at least one year is essential to adequately assess the lung damage associated with PHMG use.

Chest computed tomography (CT) is the best method for creating cross-sectional images of the chest and allows for examination of lung abnormalities. Chest CT can diagnose fibrosis or bronchiectasis, dilatation of the bronchioles due to inflammatory airway disease or fibrosis without histopathologic confirmation [10]. Additionally, chest CT is the imaging modality most commonly used to diagnose lung cancer. There has been no research with conventional chest CT to evaluate the lungs of experimental animals, and because micro-CT has been widely used instead. In micro-CT, the scan time per animal is several minutes without respiratory gating, and with respiratory gating, it is usually longer [11]. However, conventional chest CT using ventilator after intubation can obtain images in less than five seconds per animal and is easier to manage than micro-CT, as the appropriate imaging conditions can be easily adjusted. Based on these advantages, conventional chest CT images of many experimental animals can be obtained quickly.

Therefore, the purpose of this study was to evaluate long-term lung damage after PHMG exposure using conventional chest CT and histopathologic findings in the rat model.

## Materials and methods

### Animals

This study was approved by the Institutional Animal Care and Use Committee of the Korea University Medical Center (Approval number: Korea-2019-0133). All experiments were performed per the National Institutes of Health guide for the care and use of laboratory animals. Nine-week-old male Sprague-Dawley rats (Raonbio, Yong-in, South Korea) were acclimated for one week (two rats per cage). The conditions were as follows: temperature, 22–25°C; relative humidity, 40–60%; and lighting condition, light 12 hours/dark 12 hours. Pelleted food (Purina, Sung-nam, South Korea) and filtered tap water were given *ad libitum*. Each week during the experiment, weight change, appearance (rough coat, abnormal posture, or enlarged pupils), measurable clinical signs (cardiac and respiratory rates increased up to 50%, or markedly reduced), unprovoked behavior (unsolicited vocalizations, self-mutilation, very restless or immobile), and behavioral responses to external stimuli (violent reactions, or comatose) in all rats were assessed by specialized facility staff. We considered euthanizing the animals if they became debilitated, lost 20% of their body weight before the study, or if tumors interfered with walking/eating/drinking (S1 Table). However, no rat was euthanized due to the above criteria.

### Experimental design

**Fig 1:**
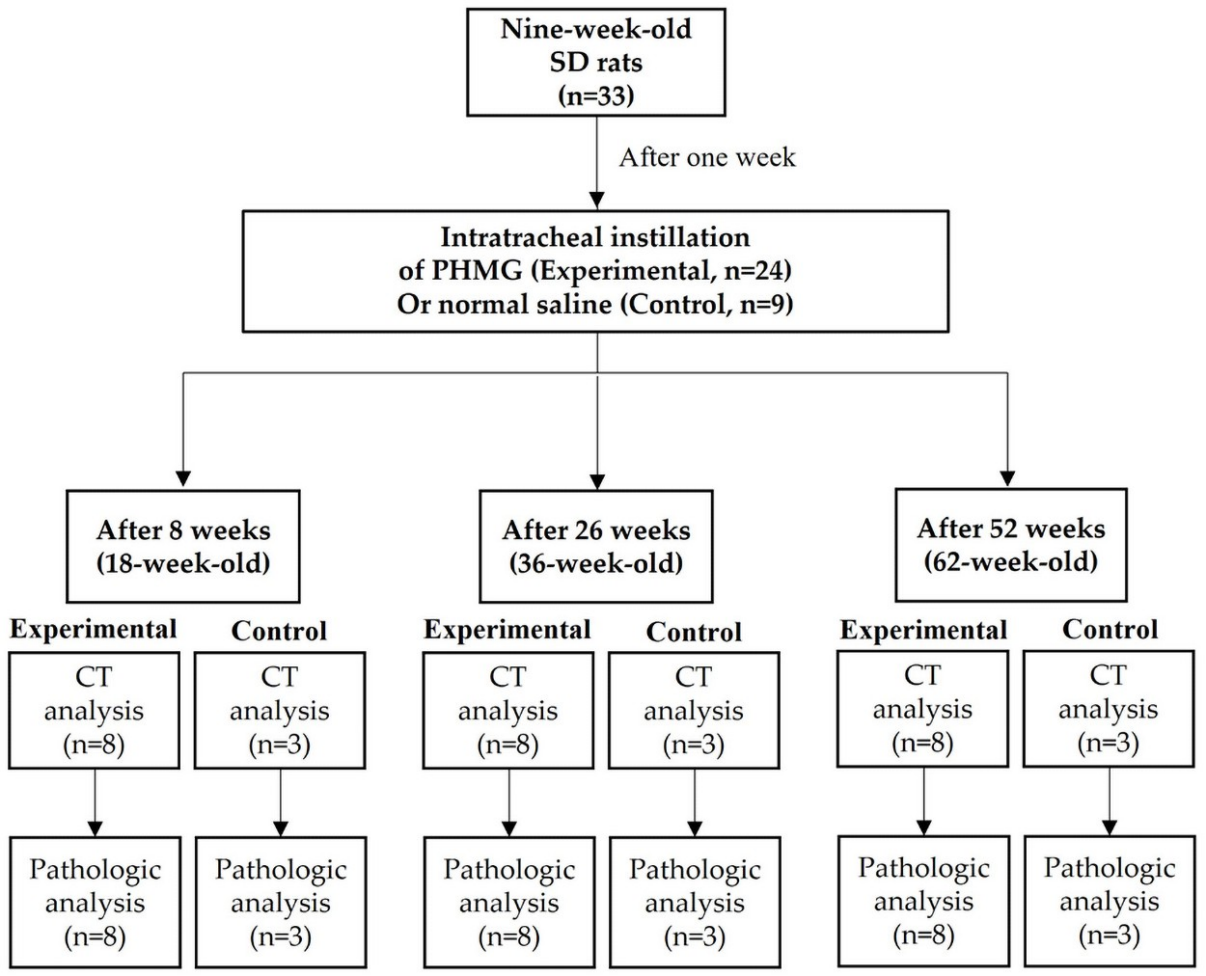
The experimental design. Conventional chest CT examination was conducted in all rats under anesthesia at 8, 26, or 52 weeks after instillation. Subsequently, the animals were sacrificed and both lungs were collected for histopathologic evaluation.

A total of 33 rats were randomly divided into three experimental and three control groups. For the experimental group, eight rats per group were allocated for eight weeks post-PHMG instillation, 26 weeks post- PHMG instillation, or 52 weeks post- PHMG instillation. For the control group, three rats per group were allocated for eight weeks post-normal saline instillation, 26 weeks post-normal saline instillation, or 52 weeks post-normal saline instillation. All rats were anesthetized with 2% isofluorane in 70% N_2_O and 30% O_2_ for intratracheal instillation of PHMG or normal saline. For the experimental group, a solution of PHMG (25% in water) was diluted to 0.9 mg/kg with saline according to previous studies, which induced a moderate degree of pulmonary fibrosis with minimal mortality [8,12,13]. Next, 50 uL of a PHMG solution was intratracheally administrated to each rat under a modified videoscope guide for intratracheal instillation. For the control group, 50 uL of normal saline was intratracheally administrated to each rat under a modified videoscope guide for intratracheal instillation. At 8, 26, or 52 weeks after PHMG or normal saline instillation, all rats underwent conventional chest CT examination under anesthetic conditions with an intraperitoneal and intramuscular injection of Alfaxan (30mg/kg) and Xylazine (10mg/kg), respectively. At the end of the protocol, rats were euthanized with CO_2_. Both lungs were then collected for histopathologic evaluation. The body weights of both the experimental and control groups are compared in S2 Table. The experimental design is summarized in Fig 1.

### Histopathologic examination

The lungs were fixed in 10% neutral buffered formalin. From the fixed samples, 4-um-thick paraffin sections were cut and hematoxylin and eosin and Masson’s trichrome staining were performed. All lung tissue was made into a slide at 3 mm intervals. The presence and number of bronchoalveolar hyperplasia and tumors were evaluated in each group. The degree of lung fibrosis was evaluated using two methods. First, we evaluated the extent (none, lesions involving <0–25%/<25–50%/>50% of the total lung areas) and severity (none/mild/moderate/severe) of the fibrosis were evaluated. The fibrosis score was calculated by adding the extent and severity. Second, a modified Ashcroft score was used to quantify fibrosis [14].

Immunohistochemical staining was used to evaluate TTF-1, p63, and CK7 expression. The sections were deparaffinized in xylene and dehydrated in a graded ethanol series followed by heat-induced epitope retrieval in a bond-epitope retrieval solution. Protein expression was detected using a primary antibody against TTF-1 (monoclonal (clone: SPT24), 1:200, Novocastra, UK), p63 (monoclonal [clone: DAK-p63], 1:100, Dako, Denmark), and CK7 (monoclonal (clone: OV-TL 12/30), 1:500, Dako, Denmark). After incubation with a bond polymer refine and 3,3′-diaminobenzidine (DAB) detection kits, the slides were rinsed and counterstained with Harris hematoxylin. Staining in the nucleus and not in the cytoplasm was considered a positive result. All extracted lung specimens were evaluated by one pathologist with 20 years of clinical experience in lung pathology (J.L.).

### CT protocol

All CT images were scanned using a Philips IQon 128-slice dual-layer detector spectral CT scanner (Philips Healthcare, Cleveland, OH, USA) with the subject in the supine position. All images were obtained in a caudo-cranial direction from the lung base through the thoracic inlet level during an inspiration breath-hold using a ventilator for small animals (VentElite, Harvard Apparatus, MA, USA). CT scan parameters were as follows: kVp, 80; mA, 400; collimation, 64 × 0.625 mm; slice thickness, 0.67 mm; beam width, 40 mm; pitch, 1.048; and rotation time, 0.4 seconds.

### CT evaluation

One board-certified radiologist with 11 years of thoracic imaging experience (C.K.), blinded to the experimental time points, reviewed all CT images. CT findings including nodules, masses, bronchiectasis, and linear densities, were evaluated. The goal of this study is to apply the results to humans. Therefore, the analysis of CT findings was performed in the same way that radiologists analyze human CT findings. Therefore these CT findings followed or modified the glossary of radiologic terms suggested by the Fleischner Society [10]. “Nodules” were defined as rounded or irregular opacities, well or poorly defined, measuring equal to or less than 1 mm in diameter. Well or poorly defined, rounded or irregular opacities of > 1 mm were defined as “masses.” “Bronchiectasis” included bronchial dilatation with respect to the accompanying pulmonary artery, with a lack of bronchial tapering. “Linear densities” were focal or multifocal subsegmental atelectasis or fibrosis showing linear configuration, almost always extending to the pleura. These CT findings were evaluated in four lobes of the right lung (the anterior, middle, posterior, and post-caval lobes) and three regions of the left lung (the upper, middle, and lower regions) according to previous research [15,16], although the Nomina Anatomanica Veterinaria divides the right lung into pars cranialis, pars caudalis, lobus medius, and lobus accessories, and the left lung into a pars cranialis and a pars caudalis [17]. As bronchiectasis and linear densities are CT findings indicative of fibrosis as in human CT, bronchiectasis and linear densities scoring were calculated by summing the presence/absence of bronchiectasis or linear densities for fibrosis analysis in each lobe or region. All CT findings were correlated with histopathologic results in consensus with radiologists and pathologists.

The volume of masses or nodules that proved lung malignancy was measured using commercially available software (Aquarius iNtuition Edition, Terarecon, Foster City, CA, USA) for nodule volumetry used in a previous study [18].

### Statistical analysis

All statistical analysis was performed by a statistical specialist (J.C.). The chi-square test for nominal variables and Kruskal–Wallis test for continuous variables were performed to determine differences between CT features and histopathologic findings among the groups. We performed a chi-square trend analysis for nominal data and a Jonckheere-Terpstra test for continuous data with Bonferroni corrections to reveal the chronologic changes of CT features and histopathologic findings. All results were presented using descriptive statistics of proportion or mean±standard deviation (SD). All statistical analyses were performed using SPSS Statistics 20 (SPSS, Chicago, IL, USA). All P-values < 0.05 were considered statistically significant.

## Results

### Characteristics of lung tumors

**Fig 2:**
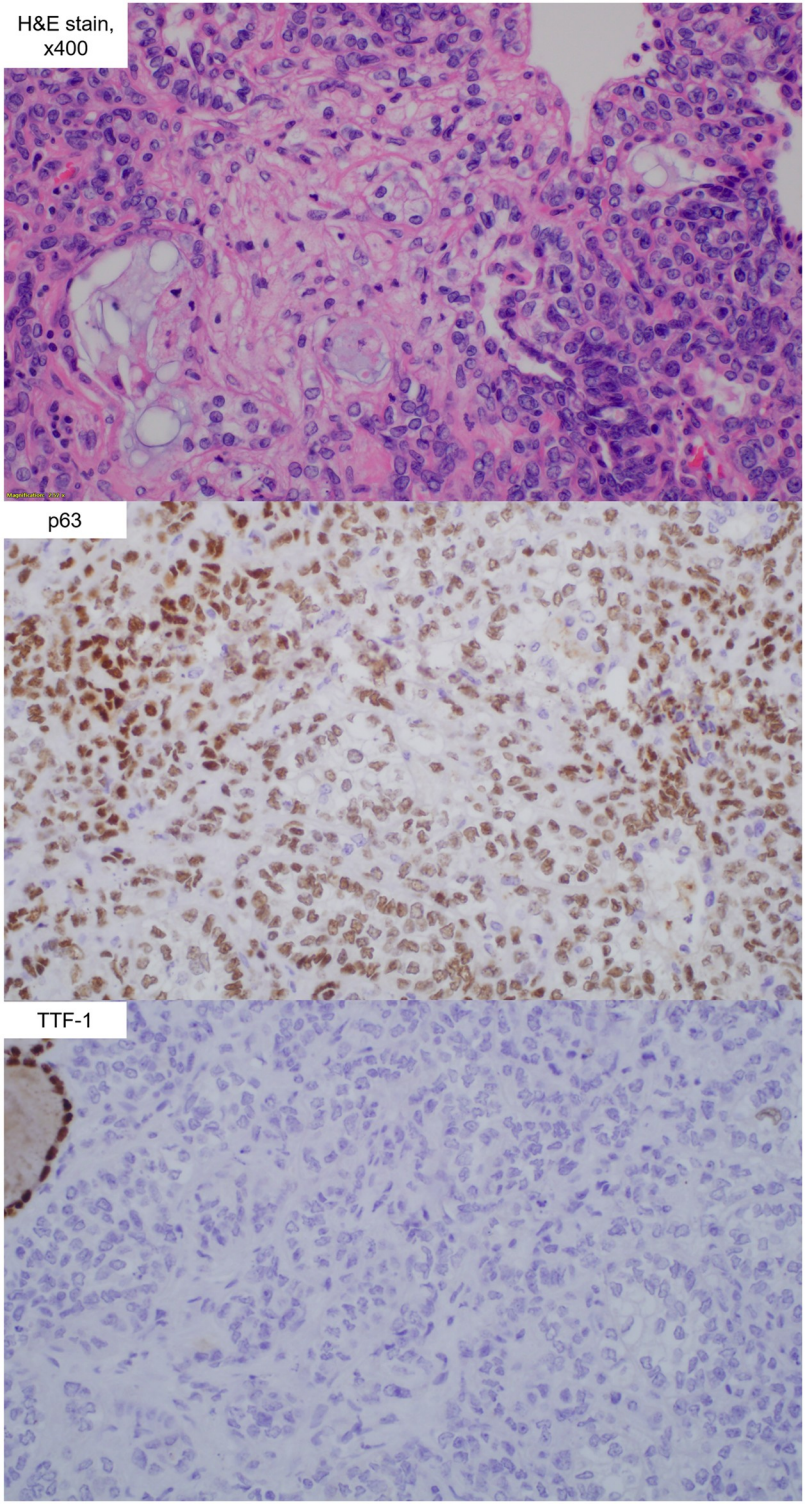
The histopathologic finding (hematoylin and eosin stain, x 400) of squamous cell carcinoma from rat #1. The squamous cell carcinoma tumor cells were positive for p63 and negative for TTF-1.

**Fig 3:**
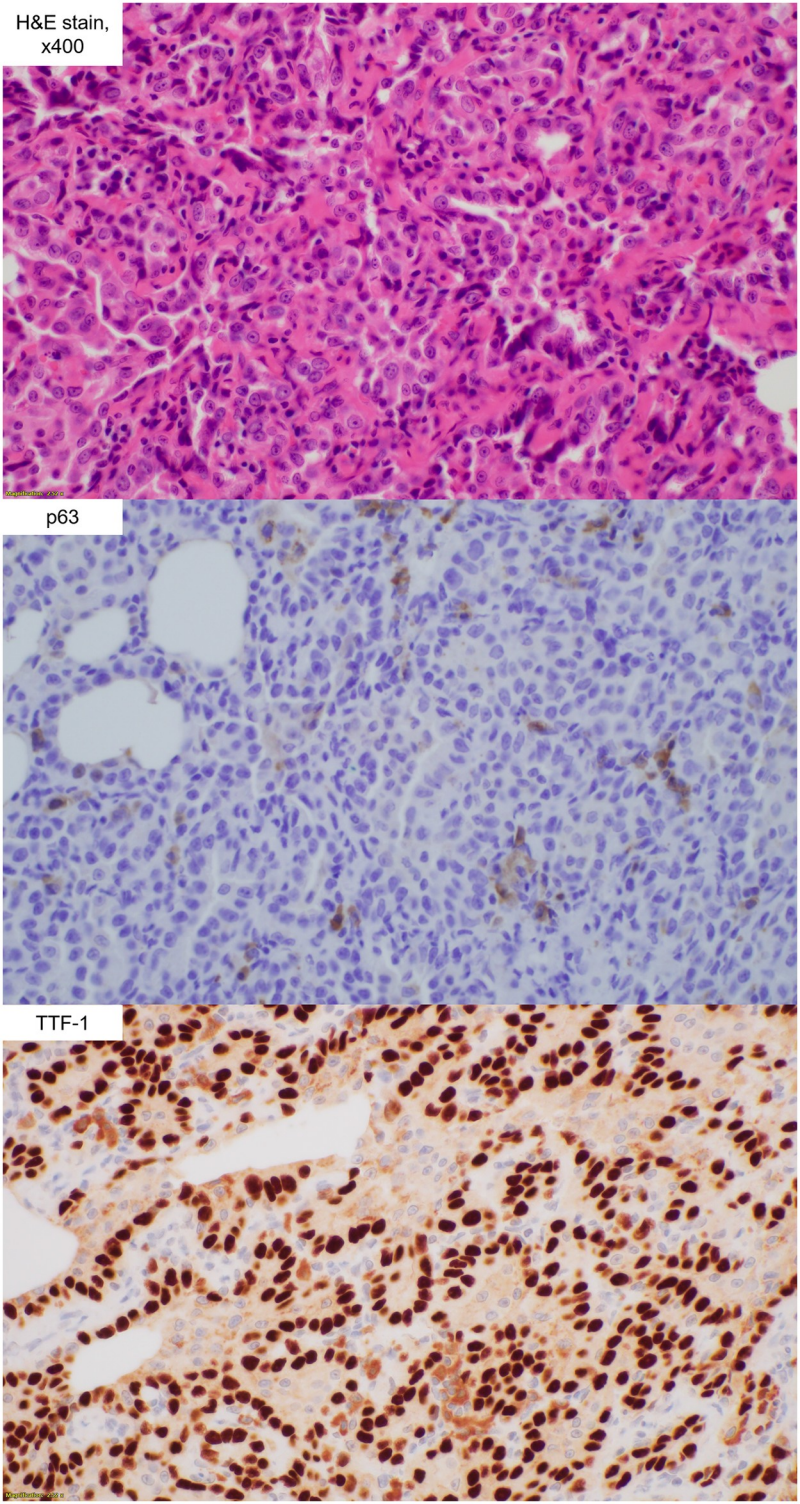
Histopathology (hematoylin and eosin stain, x 400) of adenocarcinoma. The adenocarcinoma tumor cells were positive for TTF-1 and negative for p63.

A total of four lung carcinomas, including one squamous cell carcinoma, two adenocarcinomas, and one adenocarcinoma in situ, were found in three rats (3/8, 37.5%) 52 weeks after PHMG exposure (Table 1). The size of these lung malignancies ranged from 2 to 7 mm. The largest tumor was squamous cell carcinoma. The tumor cells of the squamous cell carcinoma were positive for p63 but negative for TTF-1 and CK7 (Fig 2). In contrast, the tumor cells of the adenocarcinoma and adenocarcinoma in situ were positive for TTF-1 and CK7 but negative for p63 (Fig 3).

**Table 1:** The histopathologic characteristics of lung carcinomas 52 weeks after PHMG exposure.

Rat number	Type of cancer	Tumor volume	Largest diameter	Location
#1	Squamous cell carcinoma	0.079 mm^3^	7 mm	Left lower region
#2	Adenocarcinoma	0.016 mm^3^	3 mm	Left upper region
#2	Adenocarcinoma	0.010 mm^3^	2 mm	Left lower region
#3	Adenocarcinoma in situ	0.010 mm^3^	2 mm	Left middle region

**Fig 4:**
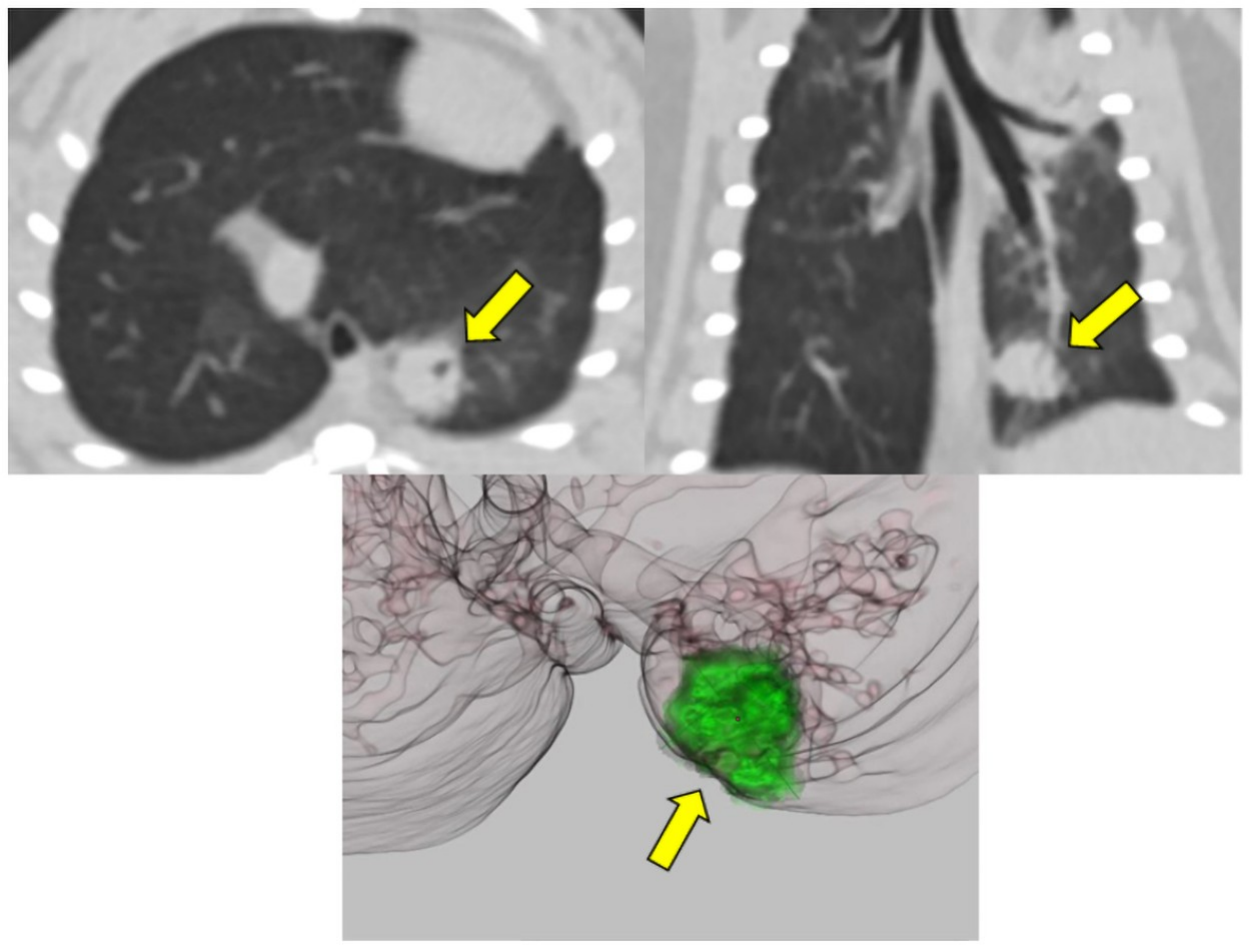
Conventional chest CT images with axial and coronal reconstruction of squamous cell carcinoma from rat #1 show a mass in the left lower region (upper panel, arrows). The nodule shows a spiculated border and bubbly appearance. These CT findings are similar to lung cancer found in humans. The volume of this squamous cell carcinoma in the 3D reconstructed image was 0.079 mm^3^, and the longest diameter was 7 mm (lower panel, arrow).

**Fig 5:**
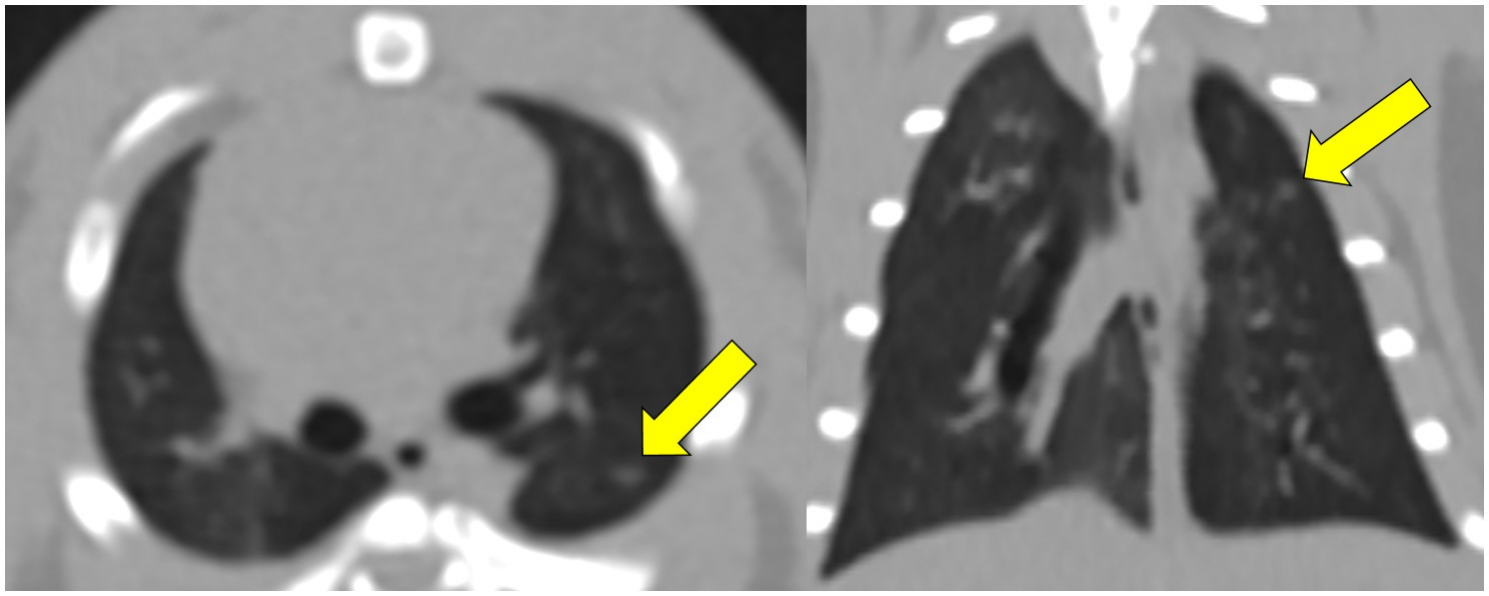
Chest CT images with axial and coronal reconstruction of adenocarcinoma from rat #2 show a nodule in the left upper region (arrow).

In the conventional chest CT, all four lung carcinomas were found and correlated with histopathologic findings (Figs 4 and 5). Conventional CT findings of a squamous cell carcinoma found in #1 rat were similar to lung cancer in humans, as they had spiculated borders and the bubbly appearance believed to represent air bronchiolograms.

### Incidence and number of lung tumors

**Fig 6:**
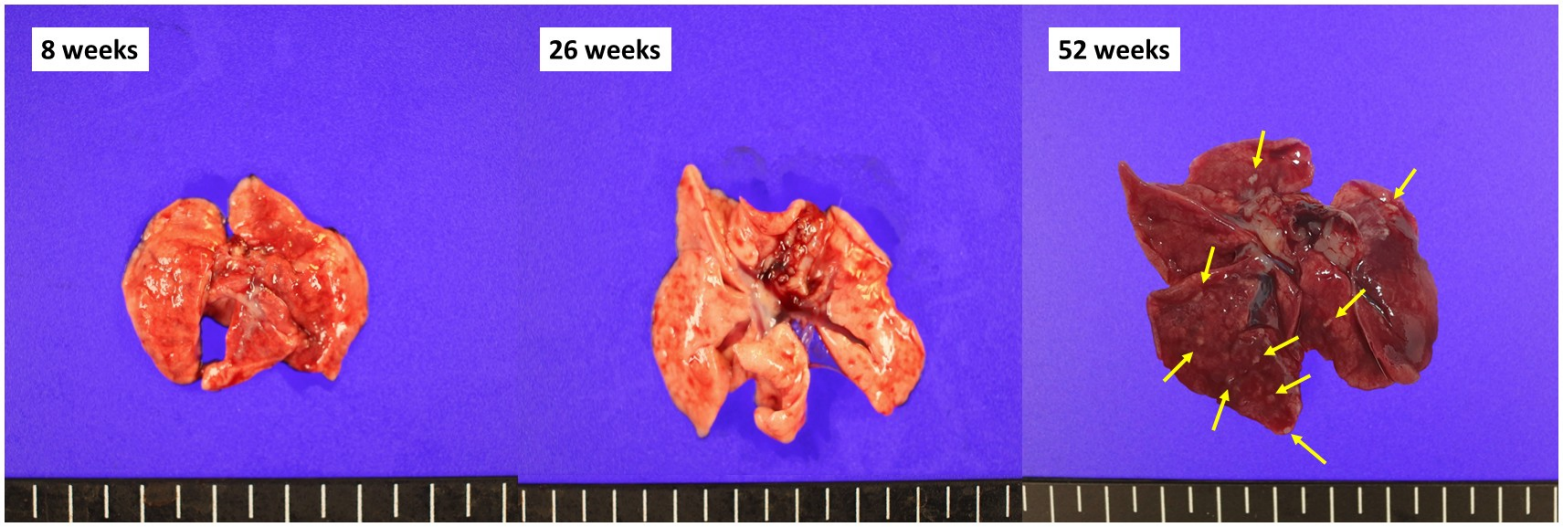
Gross pictures of extracted lungs at 8, 26, and 52 weeks-post PHMG exposure. Multiple yellowish nodules were disseminated on the surface of both lungs 52 weeks-post PHMG exposure (arrows). These disseminated nodules were bronchiolo-alveolar hyperplasia.

Table 2 shows the incidence and number of bronchiolo-alveolar hyperplasia and tumors, including bronchiolo-alveolar adenoma and carcinoma after PHMG exposure. Bronchiolo-alveolar hyperplasia was found in seven rats (87.5%) at 8 weeks and in all rats (100%) at 26 and 52 weeks after PHMG exposure. The number of bronchiolo-alveolar hyperplasia significantly increased over time after PHMG exposure (P-value for trend < 0.001) (Fig 6).

**Table 2:** The incidence and number of bronchiolo-alveolar hyperplasia and tumors after PHMG exposure.

	8 weeks	26 weeks	52 weeks	P-value	P-value for trends
*Bronchiolo-alveolar hyperplasia*					
Incidence	7 (87.5%)	8 (100%)	8 (100%)	0.352	0.221
Number of lesions per rat	8.50±8.64	18.71±16.03	54.36±17.20	**< 0.001**	**< 0.001**
*Bronchiolo-alveolar adenoma*					
Incidence	3 (37.5%)	2 (25%)	4 (50%)	0.587	0.613
Number of lesions per rat	0.50±0.55	0.29±0.49	0.64±0.67	0.516	0.579
*Carcinoma*					
Incidence	0	0	3 (37.5%)	**0.032**	0.026
Number of lesions per rat	0	0	0.36±0.67	**0.038**	0.204

Bronchiolo-alveolar adenomas were found in rats at all weeks post-PHMG exposure (37.5% at 8 weeks, 25% at 26 weeks, and 50% at 52 weeks), although there were no significant differences in incidence and numbers. Carcinomas were found only in rats at 52 weeks after PHMG exposure, with significant differences in both incidence and numbers (all P < 0.05).

In contrast, there were no bronchiolo-alveolar hyperplasia, adenomas, and carcinoma in the control group.

### Extent and severity of fibrosis

The extent and severity of fibrosis in the experimental and control groups are demonstrated in Table 3 and S3 Table. All rats showed fibrosis in less than 25% of all lung fields at 8 and 26 weeks. At 52 weeks, 25% of the rats showed fibrosis at an extent of 25–50%, whereas 75% of rats showed fibrosis in less than 25% of all lung fields. There was no difference in the extent of pulmonary fibrosis over time.

**Table 3:** The extent and severity of fibrosis after PHMG exposure.

	8 weeks	26 weeks	52 weeks	P-value	P-value for trends
Fibrosis extent					
None	0	0	0	0.113	0.077
<25%	8 (100%)	8 (100%)	6 (75%)		
25–50%	0	0	2 (25%)		
>50%	0	0	0		
Fibrosis severity					
None	0	0	0	**0.010**	**0.002**
Mild	4 (50%)	0	0		
Moderate	4 (50%)	3 (37.5%)	3 (37.5%)		
Severe	0	5 (62.5%)	5 (62.5%)		
Fibrosis score	2.50±0.54	3.63±0.52*	3.88±0.84*	**0.002**	**0.023**
Ashcroft score	2.83±0.41	3.71±0.76*	4.00±0.58*	**0.011**	**0.004**

*P < 0.05 with a comparison over eight weeks.

**Fig 7:**
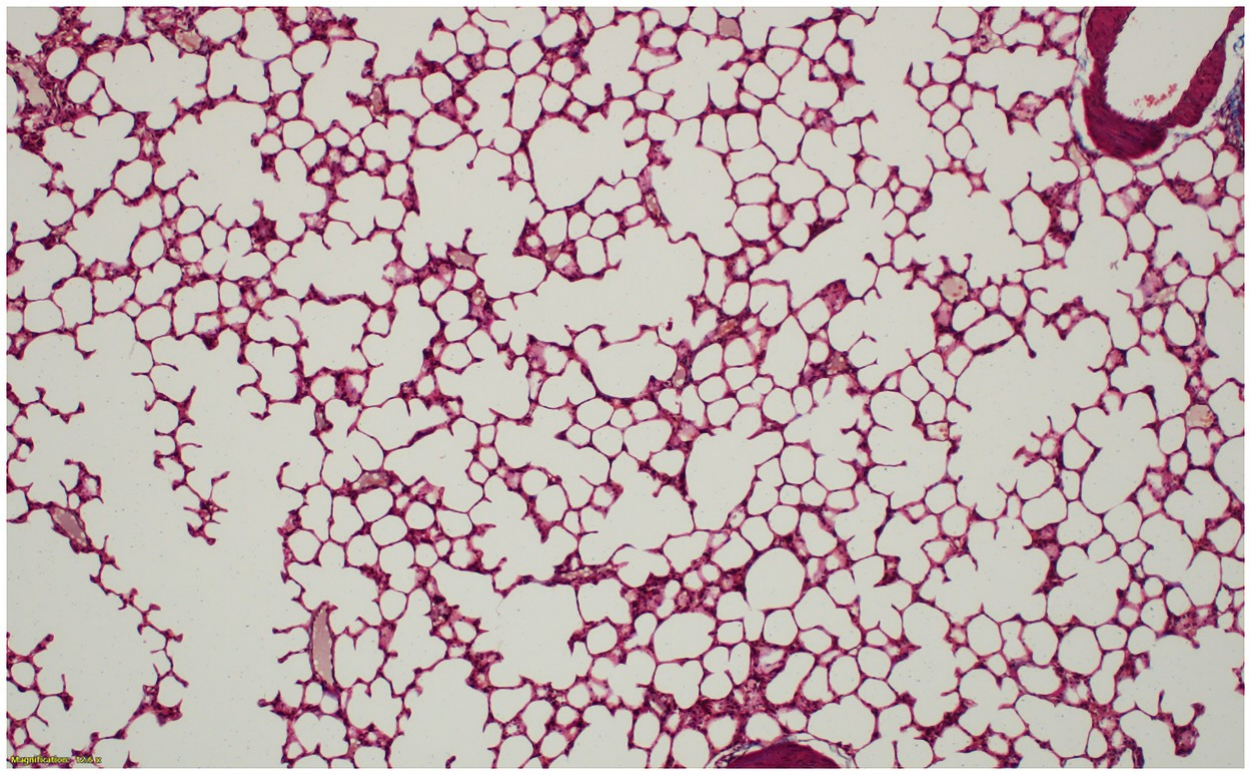
No fibrosis in control lungs at 8 weeks after normal saline instillation (Ashcroft score 0) (Masson’s trichrome (MT) stain, x100).

**Fig 8:**
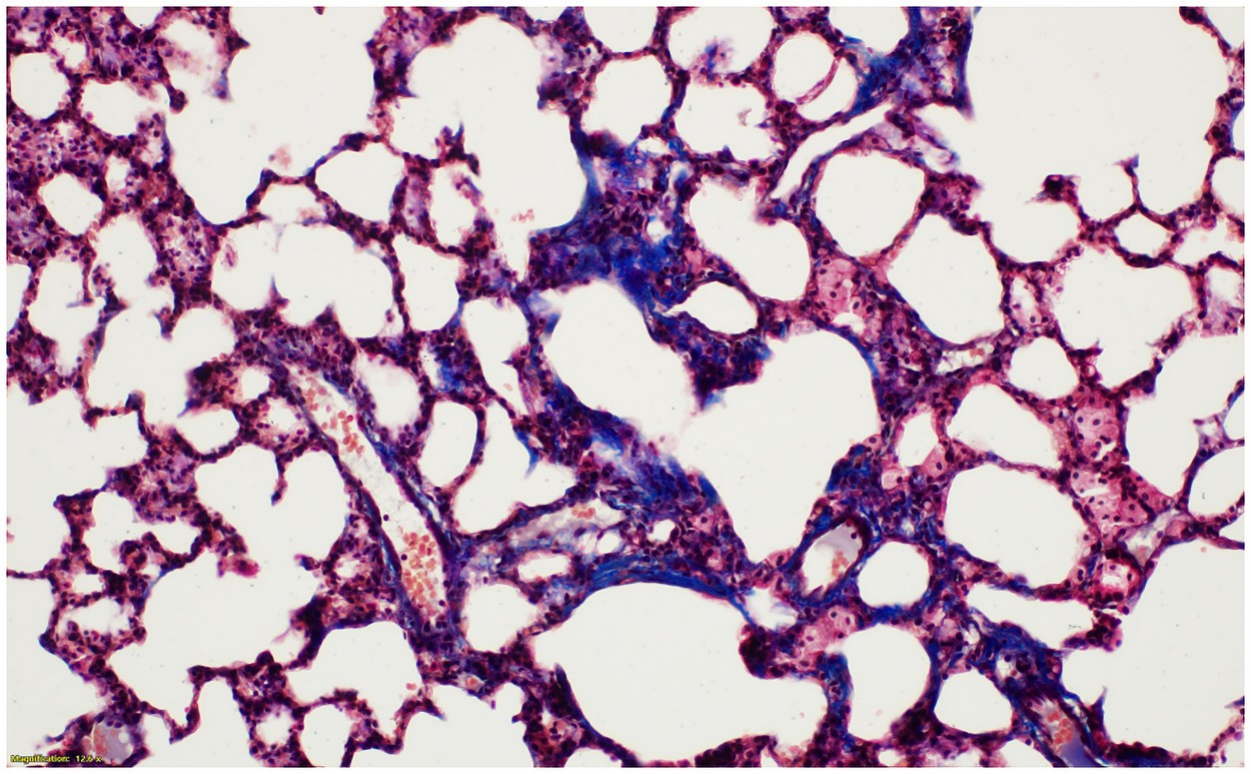
The histologic findings of lung fibrosis at 8 weeks after PHMG exposure. Contiguous interstitial thickening of alveolar septa is shown (Ashcroft score 3) (MT stain, x200).

**Fig 9:**
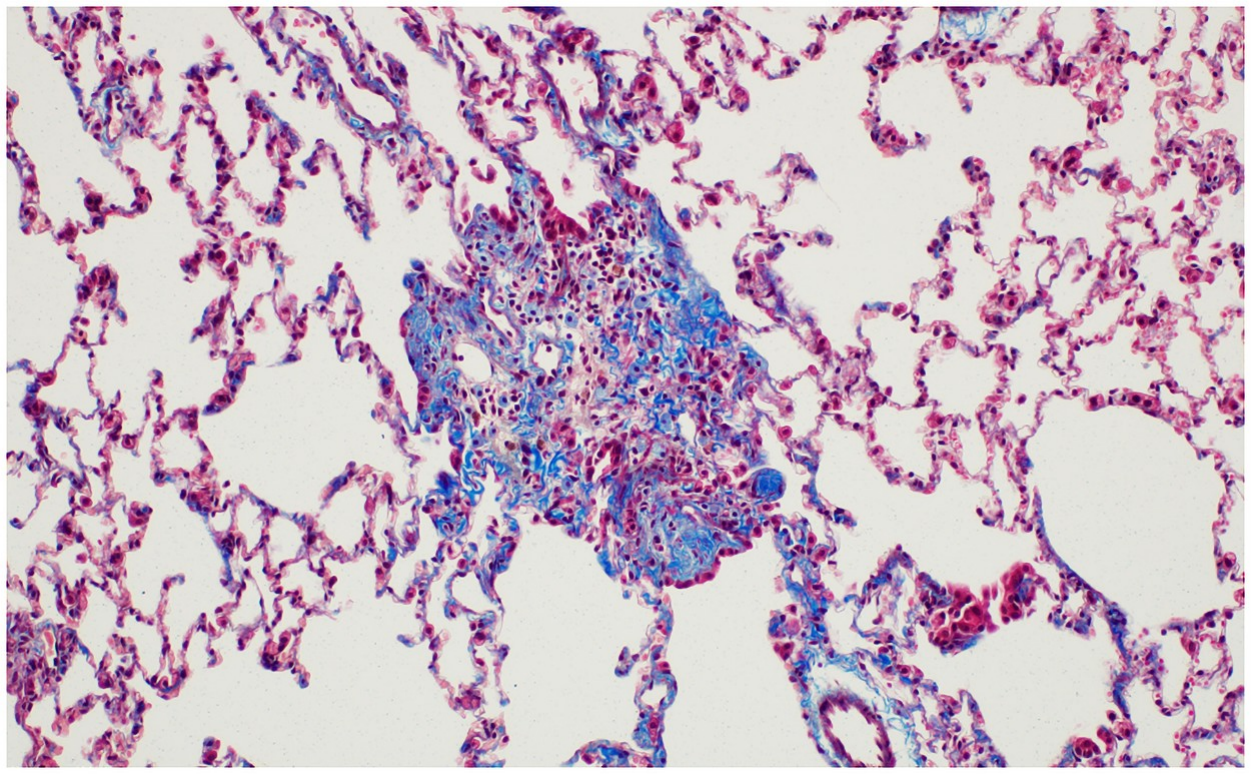
The histologic findings of lung fibrosis at 26 weeks after PHMG exposure. Fibrotic mass-like lesion of alveolar septa is shown (Ashcroft score 4) (MT stain, x200).

**Fig 10:**
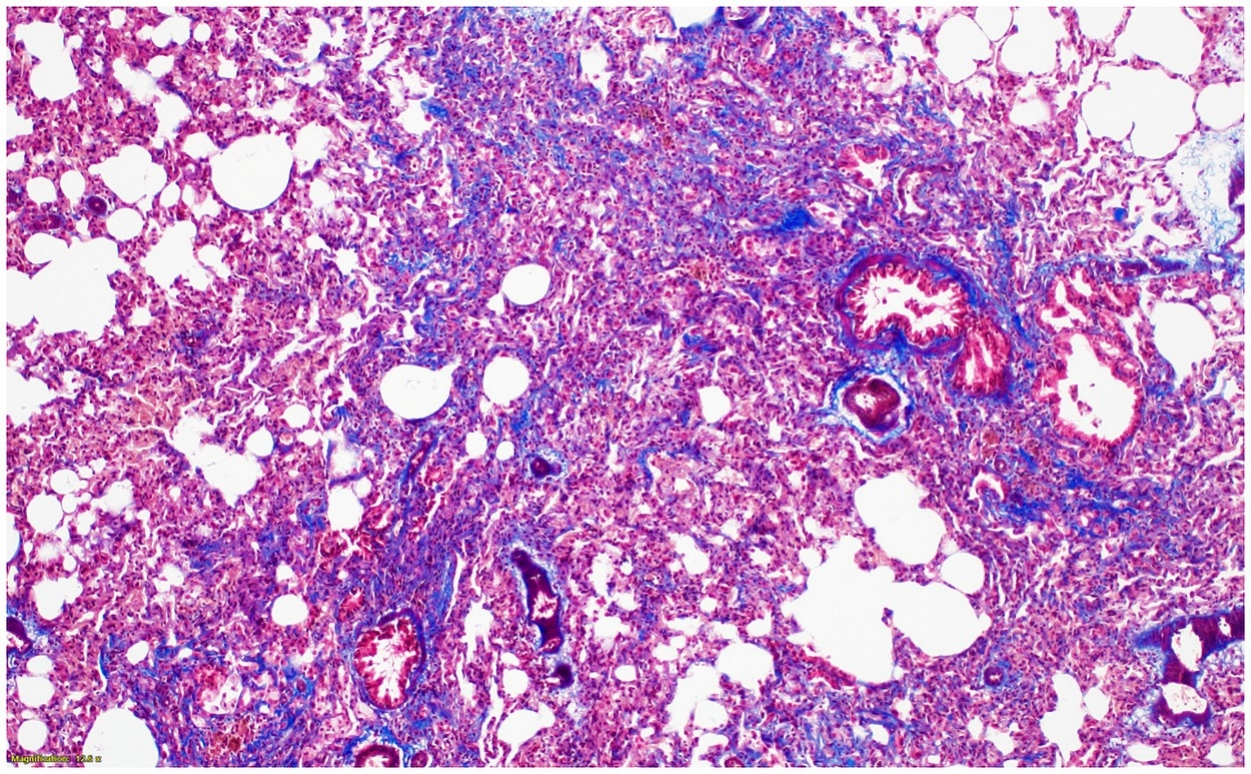
The histologic findings of lung fibrosis at 52 weeks after PHMG exposure. Increased fibrosis with definitive damage to lung structure and formation of fibrous bands are shown (Ashcroft score 5) (MT stain, x100).

However, at 26 and 52 weeks post-PHMG exposure, 62.5% of rats showed severe fibrosis and 37.5% showed a moderate degree of fibrosis. At 8 weeks, 50% of rats showed moderate fibrosis and 50% showed mild fibrosis. The severity and fibrosis scores of the pulmonary fibrosis significantly increased over time (P-values for trend = 0.002 and 0.023, respectively). Ashcroft scores also significantly increased over time (P-values for trend = 0.004). Fibrosis progressed from the peribronchiolar area to the alveolar space and often accompanied by inflammation. Destruction of the normal alveolar architecture was observed when severe fibrosis occurred (Figs 7–10).

The fibrosis extent, fibrosis severity, fibrosis score, and Ashcroft score at 8, 26, and 52 weeks after PHMG exposure were significantly higher than those of the control group (all P<0.05).

### CT image analysis

Table 4 and S4 Table provide the CT image analysis results of the experimental and control groups. All linear densities were correlated with lung fibrosis in histopathology. There were significant differences in the presence of nodules, masses, and bronchiectasis at all lobes/regions, and linear densities in the three lobes of the right lung (middle, posterior, and post-caval lobes) and the three regions of the left lung (upper, middle, and lower regions) post-PHMG exposure (all P < 0.05). The incidence of masses, bronchiectasis at all lobes/regions, and linear densities at all lobes/regions significantly increased over time after PHMG exposure (all P-values for trends < 0.05). Of the six masses found at 52 weeks, four were histopathologically confirmed lung carcinomas.

**Table 4:** Conventional chest CT image analysis 8, 26, and 52 weeks after PHMG exposure.

	8 weeks	26 weeks	52 weeks	P-value	P-value for trends
**Nodules**	8 (100%)	5 (62.5%)	8 (100%)	**0.032**	> 0.999
**Masses**	0	2 (25%)	6 (75%)	**0.005**	**0.038**
**Bronchiectasis**	3 (37.5%)	6 (75%)	8 (100%)	**0.022**	**0.007**
Right, anterior lobe	3 (37.5%)	5 (62.5%)	8 (100%)	**0.002**	**0.009**
Right, middle lobe	0	1 (12.5%)	6 (75%)	**0.002**	**0.001**
Right, posterior lobe	0	3 (37.5%)	7 (87.5%)	**0.002**	**0.001**
Post-caval lobe	1 (12.5%)	3 (37.5%)	8 (100%)	**0.002**	**0.001**
Left, upper region	0	2 (25%)	8 (100%)	**< 0.001**	**< 0.001**
Left, middle region	0	3 (37.5%)	6 (75%)	**0.008**	**0.002**
Left, lower region	0	2 (25%)	7 (87.5%)	**0.001**	**<0.001**
**Bronchiectasis score**	0.50±0.76	2.38±1.77	6.25±1.39*†	**< 0.001**	**< 0.001**
**Linear densities**	4 (50%)	6 (75%)	8 (100%)	0.069	**0.024**
Right, anterior lobe	4 (50%)	5 (62.5%)	8 (100%)	0.073	**0.031**
Right, middle lobe	0	0	7 (87.5%)	**< 0.001**	**< 0.001**
Right, posterior lobe	2 (25%)	5 (62.5%)	8 (100%)	**0.008**	**0.002**
Post-caval lobe	0	3 (37.5%)	8 (100%)	**< 0.001**	**< 0.001**
Left, upper region	2 (25%)	2 (25%)	8 (100%)	**0.002**	**0.003**
Left, middle region	0	3 (37.5%)	8 (100%)	**< 0.001**	**< 0.001**
Left, lower region	3 (37.5%)	3 (37.5%)	8 (100%)	**0.014**	**0.013**
**Linear densities score**	1.38±1.51	2.63±1.85	6.88±0.35*†	**0.002**	**< 0.001**

*P < 0.05 with a comparison of eight weeks.

^†^P < 0.05 with a comparison of 26 weeks.

**Fig 11:**
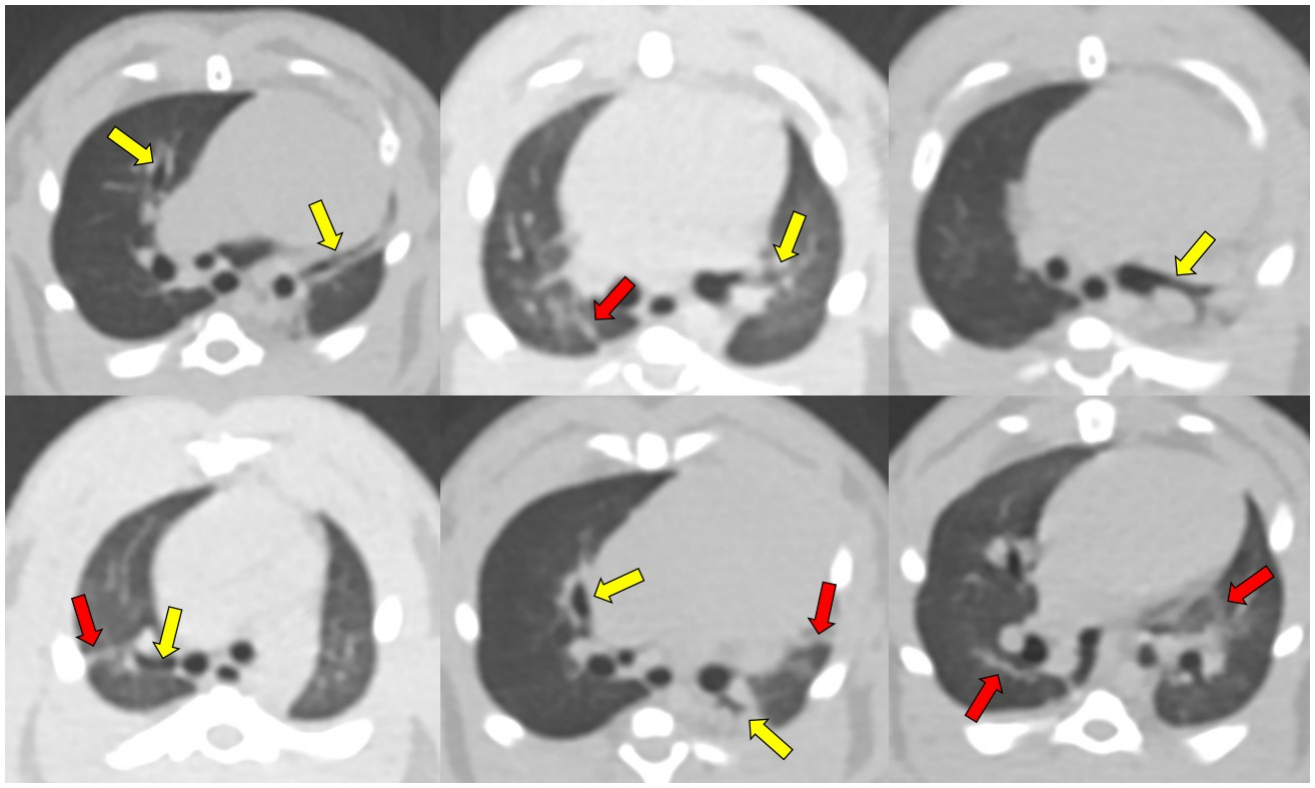
Representative CT images of bronchiectasis (yellow arrows) and linear densities (red arrows) correlated with lung fibrosis at 8, 26, and 52 weeks after PHMG exposure.

The bronchiectasis score was significantly different among the rats at 8-, 26-, and 52-weeks post-PHMG exposure (0.50±0.76, 2.38±1.77, and 6.25±1.39, respectively; P < 0.001). The bronchiectasis score at 52 weeks was significantly higher than at 8 and 26 weeks (all P < 0.05). The bronchiectasis score significantly increased over time (P-value for trends < 0.001). The linear densities score was significantly different at 8, 26, and 52 weeks post-PHMG exposure (1.38±1.51, 2.63±1.85, and 6.88±0.35, respectively; P = 0.002), and the linear densities score at 52 weeks was significantly higher than the scores at 8 and 26 weeks (all P < 0.05). The linear densities score significantly increased over time (P-value for trends < 0.001) (Fig 11).

## Discussion

This study revealed that a single instillation of PHMG resulted in lung carcinomas and many foci of bronchiolo-alveolar hyperplasia/adenomas one-year post-exposure in a rat model. Additionally, it showed increased severity of lung fibrosis over time and persistent fibrosis one year after a single exposure to PHMG. These results were supported by conventional chest CT and histopathologic analysis. It is well-established that PHMG induces lung inflammation and fibrosis in the rodent model [4–9]. However, this is the first study showing that PHMG causes lung cancer in a rodent model.

In previous research, bronchiolar-alveolar adenomas were developed 8 weeks after PHMG exposure. This study also revealed several up-regulated genes associated with lung cancer such as TOP2A and MKI67, and with tumor metastasis such as CDH11 and CD44 [12]. Bronchiolar-alveolar proliferative lesions apparently represent a spectrum that progresses from hyperplasia to adenoma to carcinoma in rodents. It has been argued that all of those lesions should be designated as carcinomas even in the earliest lesions and that bronchiolo-alveolar neoplasms are generally considered malignant in humans [19]. Based on these results, the researchers suggested future study on malignant tumor development extend beyond 8 weeks post PHMG exposure. Our experiment is a long-term follow-up study of PHMG exposure. As a result, carcinomas were found in 37.5% (3/8 rats) of the rats one year after PHMG instillation., The number of bronchiolo-alveolar hyperplasia significantly increased over time after PHMG exposure in our experiment. Based on previous study results, genetic alterations due to PHMG exposure may provoke lung injury by attenuating the normal recovery mechanism of the lung, resulting in carcinogenesis.

We suggest that the tobacco-specific carcinogen, 4-(methylnitrosamino)-1-(3-pyridyl)-1-butanone (NNK) has a similar model to PHMG, effectively inducing various lung cancers in the rodent model. Cancer began to develop after 34 weeks in mice treated with NNK, and more than half of the tumors that occurred after 54 weeks were carcinomas [20,21]. There are two major similarities between NKK and PHMG. First, a bronchiolo-alveolar hyperplasia → adenoma → carcinoma sequence was shown, and the timeline of cancer development was similar. Second, PHMG promoted DNA damage and cell cycle arrest through the ROS/ATM/p53 pathway in lung epithelial cells [22]. Our results suggest that PHMG may be a lung carcinogen, like NNK.

With CT analysis, we demonstrated that nodules and masses were significantly increased after PHMG exposure. Nodules and masses differ in size, with nodules existing at diameters of less than 1 mm and masses existing at diameters of more than 1 mm. In our study, four of the six masses found at 52 weeks were histopathologically confirmed lung carcinomas. Since all four lung carcinomas found in histopathologic analysis were 2 mm or more, the possibility of lung carcinoma is high in cases of lesions with a mass of 1 mm or more on chest CT in the rat model. In addition, CT findings of the squamous cell carcinoma were similar to lung cancer found in humans, which showed marginal spiculation and a bubbly appearance. Marginal spiculation is associated with malignancy, and several studies have confirmed marginal spiculation as a risk factor for cancer in human [23,24]. The bubbly appearance represents air bronchiolograms is also a morphologic characteristic of lung cancer on chest CT [25,26]. In humans, if a nodule shows marginal spiculation or bubbly appearance, invasive management such as tissue sampling is strongly recommended [27]. Therefore, we have shown that the occurrence of lung tumor in the rat lung model can be evaluated by conventional chest CT.

In a recent report, four patients developed lung cancers, among 113 patients with HD-related injuries (3.5%) [28]. However, the lung cancer that developed in these patients had no association with HD-related injury due to insufficient research results and a lack of medical plausibility. As the causes of lung cancer are very diverse, it is not easy to elucidate a causal relationship. In addition, since the development of lung cancer in the rodent model takes at least one year or more, a long observation period may be needed to detect lung cancer development. To our knowledge, this study is the first to reveal that PHMG may be a carcinogen in a rodent model. It supports the fact that lung cancer may occur in those with an HD-related lung injury.

We presented persistent fibrosis and increased severity of lung fibrosis one year after a single exposure to PHMG. In our experiment, the fibrosis of the lung did not disappear after one year in the rats administered PHMG, but instead became more severe. This suggests that long-term follow-up after PHMG is important because lung fibrosis caused by PHMG may be maintained for a long period. Fibrosis induced by PHMG differed from the bleomycin (BLM) rodent model usually used as a general lung fibrosis model. Previous research revealed that lung injuries induced by a single instillation of PHMG were sustained for more than four weeks, whereas lung injuries induced by a single instillation of BLM were not [7]. Several studies also reported the fibrotic lesions resolved after three to four weeks post-bleomycin [29,30]. The authors suggested that persistent fibrosis four weeks after a single instillation of PHMG might be possible due to continuous infiltration of macrophages caused by PHMG which may play a crucial role in the development of fibrosis [7]. PHMG increased the expression of fibrotic mediators and α-smooth muscle actin and induced epithelial-mesenchymal transition (EMT). In addition, pulmonary fibrosis-related micro RNAs (miRNAs) and EMT-related miRNAs were regulated by PHMG [31]. Several miRNAs associated with EMT have been identified in bleomycin-induced pulmonary fibrosis [31]. However, the cellular responses of epithelial cells against PHMG and bleomycin were not different in terms of the epithelial function associated with pulmonary fibrosis. Therefore, future investigation is needed to find the genes, miRNAs, and cytokines related to persistent lung fibrosis by PHMG.

We also provided fibrosis scoring results using conventional chest CT to evaluate lung fibrosis, including bronchiectasis and linear densities. Bronchiectasis is difficult to evaluate histopathologically but can be easily evaluated by CT. Conventional chest CT provides an overview of all lung parenchyma and facilitates a gross assessment of the overall lung pathology. While some parts of the lungs not provided as slides may be excluded from histopathologic analysis, they can be included in the CT evaluation, which allows for a better evaluation of the extent of fibrosis. In our study results, bronchiectasis and linear density scores suggestive of fibrosis increased over time and were significantly higher at one year compared with at 8 and 26 weeks post-PHMG exposure.

Several studies have reported lung fibrosis in a mouse model using micro-CT [32,33]. However, previous studies using a mouse model quantitively assessed fibrosis as lung density on micro-CT. This study assessed lung fibrosis both quantitively and qualitatively using a rat model under the guidance of a radiology expert using conventional chest CT. The qualitatively assessed lung fibrosis was also well correlated with histopathologic results.

There have been several studies for spontaneous neoplasms in aged SD rats. At 52 weeks, no tumors were found in male SD rats [34]. In another study, at 92 weeks, only one alveolar/bronchiolar carcinoma among 120 male SD rats (0.8%) was found [35]. In addition, at two years of observation, two bronchiolar alveolar adenomas (0.15%) and three bronchiolar alveolar carcinomas (0.22%) were detected among 1,340 SD rats [36]. Therefore, it is appropriate to use SD rats in a research setting, as there has been no report of lung-related tumor occurrence at 52 weeks, and the spontaneous lung tumor incidence rate is less than 1% even after 52 weeks of follow-up.

There were some limitations to this study. First, there has been no study on the precise molecular mechanism of PHMG-induced lung carcinogenesis. As the primary goal of this study was histopathologic and CT analysis after PHMG instillation, future research can focus on the molecular mechanisms. Second, in this experiment, the concentration and frequency of PHMG administration and cancer incidence were not studied.

### Conclusions

This study found that only one PHMG instillation resulted in lung cancer and irreversible fibrosis after one year, as determined by conventional chest CT and histopathologic analysis in a rat model. One year after PHMG instillation, three out of eight rats developed four carcinomas. These results suggest that PHMG may be a lung carcinogen in the rat model.

### Supporting information

S1 Table Variables for humane endpoints. (DOCX)

S2 Table The body weights of both the experimental group and control groups. (DOCX)

S3 Table The extent and severity of fibrosis in the control group (at 8, 26, and 52 weeks after intratracheal instillation of normal saline). (DOCX)

S4 Table Chest CT image analysis of the control group (at 8, 26, and 52 weeks after intratracheal instillation of normal saline). (DOCX)

S1 File Minimal data of our results. (XLSX)
