# Supplementary material for: Evaluation of the long-term effect of polyhexamethylene guanidine phosphate in a rat lung model using conventional chest computed tomography with histopathologic analysis
Source: PLoS One. 2021 Sep 7;16(9):e0256756. doi: 10.1371/journal.pone.0256756 (PMC8423271; doi:10.1371/journal.pone.0256756)
Supplement: S1 Table — (DOCX) [file pone.0256756.s001.docx]

**S1 Table**. Variables for humane endpoints.

| **Variable** | | **Score** |
| --- | --- | --- |
| **Body Weight Changes** | |  |
| 0 | Normal |  |
| 1 | < 10 percent weight loss |  |
| 2 | 10-15 percent weight loss |  |
| 3 | > 20 percent weight loss |  |
| **Physical Appearance** | |  |
| 0 | Normal |  |
| 1 | Lack of grooming |  |
| 2 | Rough coat, nasal/ocular discharge |  |
| 3 | Very rough coat, abnormal posture, enlarged pupils |  |
| **Measurable Clinical Signs** | |  |
| 0 | Normal |  |
| 1 | Small changes of potential significance |  |
| 2 | Temperature change of 1-2oC, cardiac and respiratory rates increased up to 30 percent |  |
| 3 | Temperature change of > 2oC, cardiac and respiratory rates increased up to 50 percent, or markedly reduced |  |
| **Unprovoked Behavior** | |  |
| 0 | Normal |  |
| 1 | Minor changes |  |
| 2 | Abnormal, reduced mobility, decreased alertness, inactive |  |
| 3 | Unsolicited vocalizations, self mutilation, either very restless or immobile |  |
| **Behavioral Responses to External Stimuli** | |  |
| 0 | Normal |  |
| 1 | Minor depression/exaggeration of response |  |
| 2 | Moderately abnormal responses |  |
| 3 | Violent reactions, or comatose |  |
| **TOTAL** | |  |

Reference: Morton, D.B. and P.H.M. Griffiths (1985). *Veterinary Record* 116: 431-436.
