# Supplementary material for: Evaluation of the long-term effect of polyhexamethylene guanidine phosphate in a rat lung model using conventional chest computed tomography with histopathologic analysis
Source: PLoS One. 2021 Sep 7;16(9):e0256756. doi: 10.1371/journal.pone.0256756 (PMC8423271; doi:10.1371/journal.pone.0256756)
Supplement: S2 Table — (DOCX) [file pone.0256756.s002.docx]

**S2 Table**. The body weights of both the experimental group and control groups.

|  | Experimental | Control | P-value |
| --- | --- | --- | --- |
| 8 weeks | 493.33±15.28 | 515.00±13.23 | 0.137 |
| 26 weeks | 592.14±33.93 | 619.00±1.00 | 0.222 |
| 52 weeks | 692.13±31.15 | 686.33±43.84 | 0.809 |
