# Supplementary material for: Evaluation of the long-term effect of polyhexamethylene guanidine phosphate in a rat lung model using conventional chest computed tomography with histopathologic analysis
Source: PLoS One. 2021 Sep 7;16(9):e0256756. doi: 10.1371/journal.pone.0256756 (PMC8423271; doi:10.1371/journal.pone.0256756)
Supplement: S3 Table — (DOCX) [file pone.0256756.s003.docx]

**S3 Table. The extent and severity of fibrosis in the control group (at 8, 26, and 52 weeks after intratracheal instillation of normal saline).**

|  | **8 weeks** | **26 weeks** | **52 weeks** |
| --- | --- | --- | --- |
| Fibrosis extent |  |  |  |
| None | 2 (66.6%) | 0 | 0 |
| <25% | 1 (33.3%) | 3 (100%) | 3 (100%) |
| 25-50% | 0 | 0 | 0 |
| >50% | 0 | 0 | 0 |
| Fibrosis severity |  |  |  |
| None | 2 (66.6%) | 0 | 0 |
| Mild | 1 (33.3%) | 3 (100%) | 2 (66.6%) |
| Moderate | 0 | 0 | 1 (33.3%) |
| Severe | 0 | 0 | 0 |
| Fibrosis score | 0.67±1.16 | 2.00±0.00 | 2.33±0.58 |
| Ashcroft score | 0.33±0.58 | 1.33±0.58 | 2.33±0.58 |
