# Supplementary material for: Evaluation of the long-term effect of polyhexamethylene guanidine phosphate in a rat lung model using conventional chest computed tomography with histopathologic analysis
Source: PLoS One. 2021 Sep 7;16(9):e0256756. doi: 10.1371/journal.pone.0256756 (PMC8423271; doi:10.1371/journal.pone.0256756)
Supplement: S4 Table — (DOCX) [file pone.0256756.s004.docx]

**S4 Table**. Chest CT image analysis of the control group (at 8, 26, and 52 weeks after intratracheal instillation of normal saline).

|  | **8 weeks** | **26 weeks** | **52 weeks** |
| --- | --- | --- | --- |
| **Nodules** | 0 | 0 | 0 |
| **Masses** | 0 | 0 | 0 |
| **Bronchiectasis** | 0 | 1 (33.3%) | 1 (33.3%) |
| Right, anterior lobe | 0 | 1 (33.3%) | 1 (33.3%) |
| Right, middle lobe | 0 | 0 | 0 |
| Right, posterior lobe | 0 | 0 | 0 |
| Post-caval lobe | 0 | 0 | 0 |
| Left, upper region | 0 | 0 | 0 |
| Left, middle region | 0 | 0 | 0 |
| Left, lower region | 0 | 0 | 0 |
| **Bronchiectasis score** | 0 | 0.67±0.58 | 0.67±0.58 |
| **Linear densities** | 1 (33.3%) | 2 (66.6%) | 2 (66.6%) |
| Right, anterior lobe | 1 (33.3%) | 2 (66.6%) | 2 (66.6%) |
| Right, middle lobe | 0 | 0 | 0 |
| Right, posterior lobe | 0 | 0 | 0 |
| Post-caval lobe | 0 | 0 | 0 |
| Left, upper region | 0 | 0 | 0 |
| Left, middle region | 0 | 0 | 0 |
| Left, lower region | 0 | 0 | 0 |
| **Linear densities score** | 0.33±0.58 | 1.00±0.00 | 1.00±0.00 |
